# Association of *ANRIL* Expression with Coronary Artery Disease in Type 2 Diabetic Patients

**DOI:** 10.22074/cellj.2018.4821

**Published:** 2018-01-01

**Authors:** Esmaeil Rahimi, Amirhossein Ahmadi, Mohammad Ali Boroumand, Bahram Mohammad Soltani, Mehrdad Behmanesh

**Affiliations:** 1 Department of Genetics, Faculty of Biological Sciences, Tarbiat Modares University, Tehran, Iran; 2 Tehran Heart Center, Tehran University of Medical Sciences, Tehran, Iran

**Keywords:** *ANRIL*, Coronary Artery Disease, Gene Expression, Noncoding RNA, Type 2 Diabetes Mellitus

## Abstract

**Objective:**

*ANRIL* is an important antisense noncoding RNA gene in the INK4 locus (9p21.3), a hot spot region associated with multiple disorders including coronary artery disease (CAD), type 2 diabetes mellitus (T2DM) and many different types of cancer. It has been shown that its expression is dysregulated in a variety of immune-mediated diseases. CAD is a major problem in T2DM patients and the cause of almost 60% of deaths in these patients worldwide. The aim of the present study was to compare the expression level of *ANRIL* between T2DM patients with and without CAD.

**Materials and Methods:**

In this case-control study, we examined *ANRIL* expression in peripheral blood mononuclear cell samples by quantitative reverse transcriptionpolymerase chain reaction (RT-qPCR) in 64 T2DM patients with and without CAD (33 CAD+ and 31 CADpatients respectively, established by coronary angiography).

**Results:**

Expression analysis revealed that *ANRIL* was up regulated (2.34-Fold, P=0.012) in CAD+ versus CAD diabetic patients. Data from receiver operating characteristic (ROC) curve analysis has shown that *ANRIL* could act as a potential biomarker for detecting CAD in diabetic patients.

**Conclusion:**

The expression level of *ANRIL* is associated with presence of CAD in diabetic patients and could be considered as a potential peripheral biomarker.

## Introduction

Long noncoding RNAs (lncRNAs) are one of the most important classes of RNA molecules, receiving extensive attention as potentially novel biological regulators. Manyroles have been attributed to lncRNAs including nuclear organization, dosage compensation, epigenetic modificationand RNA splicing (1, 2). Accumulated evidence has shownthat lncRNAs can exert their regulatory function in both cis and trans patterns (3). It has also been suggested thatderegulation of lncRNAs, as a key regulator of normal cellfunction, is correlated with different types of human disorders. For example, the *HOX* antisense lncRNA, *HOTAIR*, is one of the most well-known lncRNAs, with elevated expressionlevels in many cancers of tissues such as gastric, bladder and breast (4-6). 

Genome-wide association studies (GWAS) have revealed that the 9p21 locus is associated with several diseases, including CAD, T2DM and several types of cancer (7). This locus overlaps with the well- characterized lncRNA *ANRIL* [a.k.a. *CDKN2B* antisense RNA1 (*CDKN2B-AS1*)]. *ANRIL* is transcribed as a 3.8kb lncRNA in the opposite direction of the *INK4/ ARF* locus. It has been reported that *ANRIL* can directly recruit PRC2 complexes to this locus and repress the *p15/ CDKN2B-p16/CDKN2Ap14/ARF* gene cluster (8). More recent GWA studies have shown that genetic variation (SNPs) in *ANRIL* are associated with a wide variety of metabolic and immune-mediated diseases such as CAD, however, little is known regarding its molecular role in the pathology of these diseases (9, 10). For instance, it has only been shown that up-regulation of *ANRIL* affects the expression of genes related to inflammation (11).

T2DM is a well-recognized cause of multiple complications including retinopathy, nephropathy and coronary artery disease (CAD) (12-14). Atherosclerosis is the leading cause of morbidity and mortality of T2DM patients. Prevention of CAD morbidity and mortality in patient with T2DM has therefore become a major health issue worldwide (15). Given that T2DM and atherosclerosis are two closely linked disorders, many efforts have been carried out to elucidate their common etiology. Risk factors including abdominal obesity, insulin resistance and inflammation are involved in these diseases (16, 17). 

As a genomic hotspot of CAD and T2DM, we aimed to examine the expression profile of *ANRIL* in CAD+ versus CADpatients with T2DM to identify whether *ANRIL* could be a potential target for treatment or biomarker to identifying T2DM patients with CAD. 

## Materials and Methods

The subjects of this case-control study were 64 patients who had undergone coronary angiography at the Tehran Heart Center, Iran. Patients were screened for the presence of diabetes [fasting blood sugar (FBS)≥126 mg/dl (6.9 mmol/L) and/or HbA1c≥6.5%] and those who qualified as diabetic were included in the study. T2DM patients were then divided into two groups (33 CAD+ patients and 31 CAD- patients). According to the results of coronary angiography, diabetic patients with coronary artery stenosis (≥50%) were chosen as CAD+ and further classified into single-vessel disease (SVD, n=11) and multi-vessel disease (MVD, n=22) sub-groups. Also, high-density lipoprotein (HDL)cholesterol and triglyceride levels were assessed and low- density lipoprotein cholesterol level in plasma was measured by Friedewald’s formula. All subjects gave informed written consent to participate in the study. This study was approved by the Ethics Committees of Tehran Heart Center and Tarbiat Modares University. 

### Blood collection and peripheral blood mononuclear cells isolation 

Whole blood was collected from patients on the day of coronary angiography. All patients were informed not to take any food and medication for at least 12 hours before blood collection. Peripheral blood mononuclear cells (PBMCs) were immediately isolated by centrifugation by the Ficoll-Paque^TM^ (lympholyte, Cedarlane, Netherlands) gradient according to the manufacturer’s instructions. 

### RNA extraction and cDNA synthesis

The acid guanidinium-phenol-chloroform method with the RNX^TM^-Plus reagent (SinaClon Co., Iran) was used to extract total RNA from isolated PBMCs. The integrity and quality of total RNA was assessed by agarose gel electrophoresis, and its concentration was examined by spectrophotometry at 260 nm. After treatment with DNase I (Fermentas, Lithuania), to eliminate DNAcontamination, 3 µg of total RNA was used to synthesize complementary DNA (cDNA) by using random hexamer and oligo (dT)18 primers along with the M-MulV reverse transcriptase (Thermo Scientific, USA) in a total reaction volume of 20 µl, according to the manufacturer’s instructions. 

### Quantitative real-time polymerase chain reaction

Quantitative real-time polymerase chain reaction (qPCR) was undertaken in an ABI StepOne™ (Applied Biosystems, Foster City, CA, USA) machine. The expression of *ANRIL* at the transcript level was examined by using specific primers (F: GCCTCATTCTGATTCAACAGCAGAG, R: CACCTAACAGTGATGCTTGAACCC, final concentration of 4 pmol/µl for each one), 10 ng of cDNA template and 5X EvaGreen® qPCR Mix Plus (ROX) (Solis BioDyne, Estonia) in a final reaction volume of 20 µl. The thermal cycling conditions were an initial denaturation at 95°C for 10 minutes, followed by 40 cycles of denaturation at 95°C for 15 seconds, annealing at 60°C for 30 seconds and extension at 72°C for 30 seconds. Poly acrylamide gel electrophoresis and dissociation curve analysis was used to verify the specificity of the PCR product. To normalize the expression of *ANRIL, ACTB:*

F: AGCCTTCCTTCCTGGGCATGG, 

R: AGCACTGTGTTGGCGTACAGGTC

was used as an internal control. All of the samples were runin triplicate and the normalized expression levels were usedfor further analysis. The level of differential expression was calculated by the 2^-ΔΔCt^ method (12). 

### Statistical analysis 

Data were shown as mean ± SEM and analyzed fornormality with the Shapiro-Wilk test. Mann-Whitney U-testwas used to assess the statistical significance of the differentialgene expression between CAD+ and CADpatient groups. Chi-square test, Student’s t test or Mann-Whitney U test wereperformed to compare demographic variables between CAD- versus CAD+ patients Pearson correlation coefficient was usedassess the correlation of *ANRIL* expression with glycemicand lipid profiles. A P<0.05 was considered significant. All statistical tests were carried out in either SPSS (SPSS, Chicago, IL, USA, version. 18.0) or Graphpad Prism version 6.0 (Graphpad Prism Software, Inc., San Diego, CA). 

## Results

### *ANRIL* expression in the peripheral blood mononuclear cells of patients 

**Fig.1:**
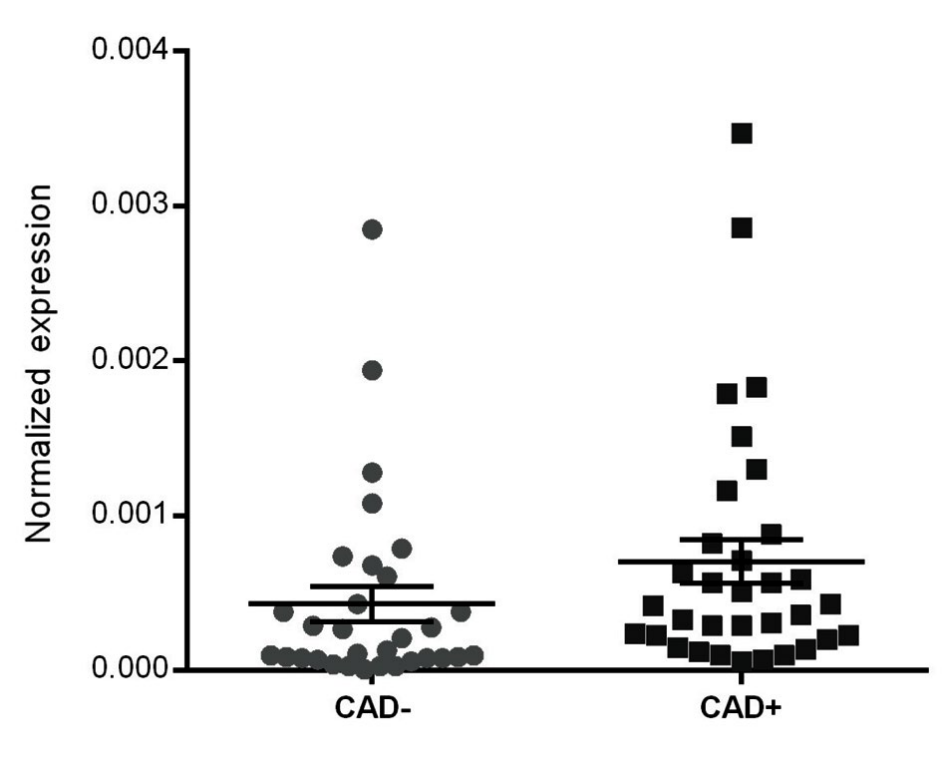
Expression level of *ANRIL* in isolated PBMCs from T2DM patients (31CADversus 33 CAD+). Expression of *ANRIL* was significantly up-regulated inCAD+ patients (Mann–Whitney U test, P<0.05). ACTB was used as an internal control for normalization. Error bars represent SEM (P=0.012). PBMCs; Peripheral blood mononuclear cells and CAD; Coronary artery disease.

**Fig.2:**
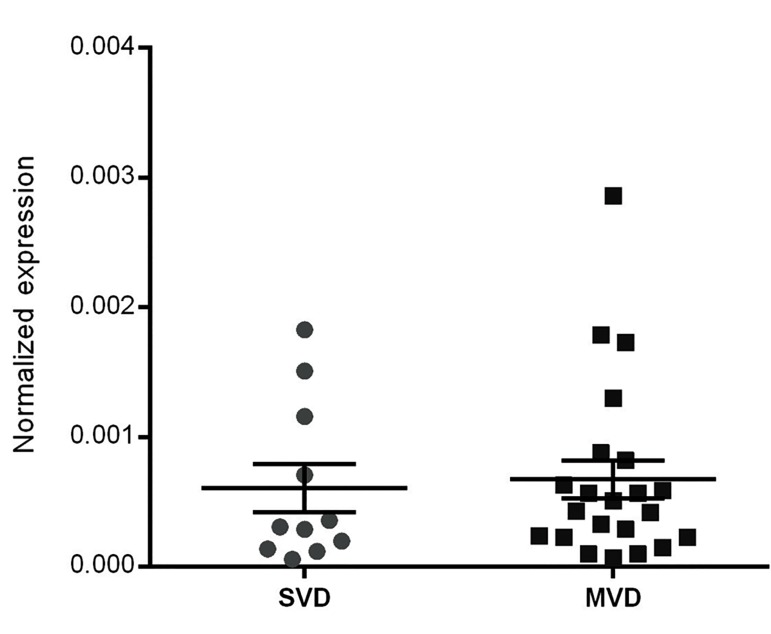
Expression level of *ANRIL* between the two CAD+ subgroups (SVD; n=11and MVD; n=22). The difference between SVD and MVD patients was notsignificant (Mann–Whitney U test, P>0.05). Error bars represent SEM. CAD; Coronary artery disease, SVD; Single-vessel disease, and MVD; Multi-vessel disease (P=0.64).

The expression of *ANRIL* was significantly up-regulatedin the CAD+ group (fold change=2.28, P=0.012) (Fig .1). This suggests that the expression of *ANRIL* is associated with atherosclerosis susceptibility in T2DM patients. Toassess whether disease severity is also associated with *ANRIL* expression level, CAD+ individuals with SVD (n=11) werecompared with those with MVD (n=22). No statisticallysignificant difference was observed between the two subgroups for the expression level of *ANRIL* (Mann-Whitney U test, P>0.05, Fig .2). 

### Effect of glycemic control and lipid profile on the expression level of *ANRIL*

Next, we examined whether glycemic control or the lipid profile of patients is related to the expression of *ANRIL* (Table 1). Analysis of the biochemical data of patients revealed that poor glycemic control may be a risk factor for the development of CAD in T2DM patients (P<0.019). We therefore examined the correlation of RNA expression of *ANRIL* with HbA1C and FBS levels by calculating the Pearson correlation coefficient test. Results showed that RNA expression of *ANRIL* was not correlated with glucose levels in these patients (r=-0.027, P=0.835). Also, the lipid profile of patients was not correlated with *ANRIL* expression (Table 2). 

### *ANRIL* as a potential biomarker for progression of atherosclerosis in T2DM 

**Fig.3:**
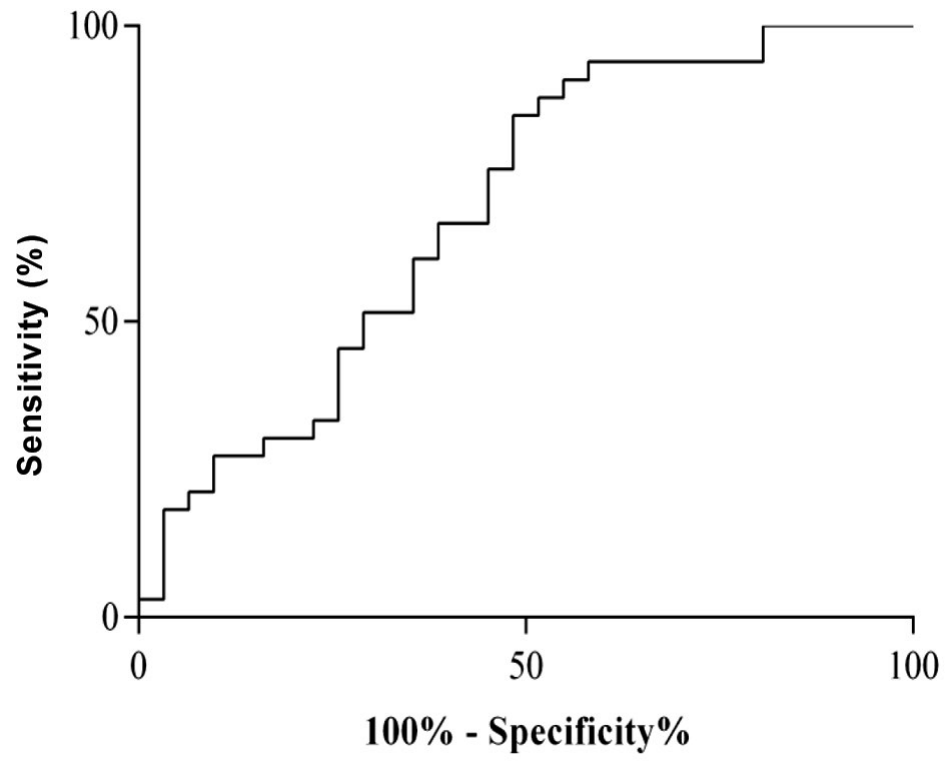
Result of ROC curve analysis for *ANRIL* expression as a potential biomarker. ROC; Receiver operating characteristic and AUC; Under the ROC curve. (AUC=0.68, P=0.012).

Receiver operating characteristic (ROC) curve analysis was performed and the area under the ROC curve (AUC) was calculated to examine whether *ANRIL* expression can be used as biomarker for identifying T2DM patients with CAD. Given that the AUC value was 0.6808 [95% confidence interval (CI): 0.5474-0.8142, P=0.012], *ANRIL* could be a potential biomarker for CAD progression (Fig .3).

**Table 1:** Clinical and demographic parameters of the patients


Characteristic	CAD n=33 (100%)	non-CAD n=31 (100%)	P values

**Age (Y)**	60.76 (9.093)	61.10 (8.047)	0.875^*^^*^
**Sex (male, %)**	60.60	48.38	0.326^*^
**BMI (kg/m^2^)**	29.19 (5.10)	27.94 (3.89)	0.554^*^^*^^*^
**Diabetes duration(months)**	95.12	100.94	0.732^*^^*^^*^
**Triglycerides (mg/dl)**	15406.55	15248.89	0.386^*^^*^^*^
**HDL (mg/dl)**	1581.97	1641.53	0.591^*^^*^
**LDL (mg/dl)**	4011.21	3682.52	0.296^*^^*^
**TCH (mg/dl)**	6619.53	6237.08	0.070^*^^*^^*^
**HbA1C**	8.45 (1.84)	7.76 (1.27)	0.019^*^^*^^*^
**Hyperlipidemia (%)**	85	81	0.656^*^
**Hypertension (%)**	76	77	0.875^*^
**Current smoking (%)**	12.12	16.13	0.645^*^
**Treatment**			
**Metformin (%)**	91	94	0.694^*^
**Glibenclamide (%)**	33	23	0.339^*^
**Statin (%)**	97	90	0.272^*^
**Insulin (%)**	12	6	0.437^*^


Data are mean ± SD or number of subjects (%). BMI; Body mass index, CAD; Coronary artery disease, HDL; High density lipoprotein, LDL; Low density lipoprotein, TCH; Total cholesterol, HbA1C; Glycated hemoglobin, *; Chi-square test, **; Student’s t test, and ***; Mann-Whitney U test were performed to compare variables between CADversus CAD+ patients.

**Table 2:** Correlation between the expression level of ANRIL with HbA1C, FBS and the lipid profiles


Correlation with	r*	P value

HbA1C	-0.027	0.835
FBS	-0.137	0.281
HDL	0.010	0.934
LDL	-0.033	0.795
Triglycerides	-0.227	0.071
TCH	-0.038	0.766


*; Pearson correlation coefficient, HbA1C; Glycated hemoglobin, FBS; Fasting blood sugar, HDL; High density lipoprotein, LDL; Low density lipoprotein, and TCH; Total cholesterol.

## Discussion

Currently extensive research is undertaken regarding lncRNA as potential biomarkers and has become one of the most popular areas in molecular medicine. Association of lncRNAs with inflammatory diseases, such as atherosclerosis and T2DM, has been discovered recently. The remarkable change in lncRNA expression in inflammatory diseases such as CAD seems to be a feature shared among some lncRNAs, rendering them as potential biomarkers as well as therapeutic targets (17, 18). However, only a few lncRNAs including the metastasis-associated lung adenocarcinoma transcript 1 *(MALAT1), HOTAIR, ANRIL*, and *lincRNA-p21* are known to be associated with human diseases (19-21).

*ANRIL* is a well-known functional lncRNA associated with multiple human diseases, particularly inflammatory diseases such as atherosclerosis (11). Given that dysregulation of *ANRIL* is associated with many diseases, *ANRIL* can be considered as a potential biomarker and therapeutic target (22). Concerning the potential role of *ANRIL* in CAD and T2DM, and its expression in inflammatory (9, 10) cells provoked us to know whether its expression in PBMCs is associated with CAD progression in T2DM patients. In this study, we found that the expression of *ANRIL* was up- regulated significantly in CAD+ diabetic patients. 

This up-regulation might be associated with the progression of CAD in T2DM patients. Holdt et al. (10) showed that expression of *ANRIL* was up-regulated in PBMCs of atherosclerosis patients and its expression was associated with severity of atherosclerosis, however, we did not observed an association with severity (SVD vs MVD patients). This inconsistency may be related to other genetic or environmental factors influencing the progress of atherosclerosis disease in our population. In the case of other genetic factors, whole genome analysis will be informative. Since the rate of atherosclerosis in T2DM patients is high, we highlight the importance of predicting atherosclerosis in these patients. However, this was a preliminary study in this case and further investigation is required to confirm *ANRIL* expression level as a biomarker for predicting atherosclerosis in T2DM patients. 

What might be the role of *ANRIL* in the progression of atherosclerosis in diabetic patients? It is well-known that *ANRIL* regulates the expression of protein-coding genes by recruiting Polycomb repressive complexes to their promoter (23, 24). Zhou et al. (11) also showed that *ANRIL* up-regulates the expression of many inflammatory genes. In addition, many studies have shown that atherosclerosis and T2DM are chronic inflammatory diseases (25, 26). It is thus possible that *ANRIL* regulates the expression of inflammatory genes. Lack of association of *ANRIL* expression with high glucose and lipids profile is also consistent with its major role in inflammation. 

## Conclusion

We show that the association of the 9p21 locus with CAD in T2DM patients is likely to be due to *ANRIL* by dysregulating neighboring or inflammatory genes. However, to confirm this claim, further mechanistic studies are required to know whether *ANRIL* is a cause of CAD in T2DM patients or an associated biomarker.
